# Reengineering Vital Registration and Statistics Systems for the United States

**Published:** 2004-09-15

**Authors:** Charles J. Rothwell

**Affiliations:** National Center for Health Statistics, Centers for Disease Control and Prevention

## Abstract

For more than a hundred years, the United States has operated a decentralized vital statistics system as an essential component of public health. Statistics based on births and deaths registered in the United States are a primary source of data used to track health status, to plan, implement, and evaluate health and social services, and to set health policy. The national vital statistics system provides nearly complete, continuous, and comparable federal, state, and local data. The system, however, is based on outmoded vital registration practices and structures, which raises concerns about data quality, timeliness, and the lack of real-time linkage capabilities. While many organizations are working together to address these issues and have made notable achievements, questions remain to be answered. Efforts to rejuvenate the nation's vital statistics system will need to expand dramatically to provide public health with a timely, high-quality, and flexible system to monitor vital health outcomes at the local, state, and national levels.

## Essay

For more than a hundred years, the United States has operated a decentralized vital statistics system as an essential component of public health. Statistics based on births and deaths registered in the United States are a primary source of data used to track the health status of the U.S. population, to plan, implement, and evaluate health and social services for children, families, and adults, and to set health policy at the national, state, and local levels. Data on access to prenatal care, maternal risk factors, infant mortality, disparities in health status, changes in the rankings of causes of death, life expectancy, years of potential life lost, and other pregnancy and mortality indicators provide the staples for public policy and programmatic debates about improving health and health services delivery. Unlike any other public health data system, the national vital statistics system provides nearly complete, continuous, and comparable federal, state, and local data to public health officials and programs. This strength enables population-based analysis and comparisons to be undertaken at the national, state, and local levels by age, race, ethnicity, and sex. For example, with more than two million deaths each year, disparities in the leading causes of death by race and age can be monitored and compared at the local, state, and national levels. Rare and emerging causes of death can be identified, and using both the underlying and contributing causes of death, the impact of such diseases as hypertension, diabetes, and atherosclerosis on mortality can be measured.

Despite the importance of the nation's vital statistics system, it is based on outmoded vital registration practices and systems, a fact that raises concerns about data quality, timeliness, and the lack of real-time linkage capabilities for the more than six million annual vital events. To resolve these issues, vital registration requires more complete automation at the level of primary data collection and changes in the relationships among the providers of source records, the state registration offices, and the National Center for Health Statistics (NCHS). For example, for almost 20 years, states have been using electronic birth certificate systems. While this is a significant step forward, states continue to operate dual paper and electronic systems, with the paper record considered the official legal document. To compound these problems, the current electronic systems for vital registration at the state level have been difficult to modify, causing many states to delay implementation of the 2003 revisions to the U.S. standard certificates, which would provide a wealth of new information. Collection of death information continues to be primarily a paper-based process, unchanged at the local and state levels for the last half century. Funeral directors are responsible for collecting demographic information on the decedent from the next of kin, while attending physicians, medical examiners, or coroners provide and certify medical information on cause of death. Demographic and medical information are brought together manually by passing the paper certificate back and forth; the certificate data does not become computerized until reaching the state vital registration office, sometimes after considerable delay. The lack of automation at the source precludes timely follow-back to improve data quality and does not take advantage of existing internal systems of funeral directors and physicians. The Internet is not even used for electronic data transfer between data providers and state registration offices.

To address these problems, the National Association of Public Health Statistics and Information Systems, NCHS, and the Social Security Administration have developed a partnership to improve the responsiveness of state vital registration and statistics systems. Their objective is to improve the timeliness, quality, and sustainability of these systems by adopting national, consensus-based standards and guidelines. It will be necessary to go beyond modifying existing registration systems. State processes and systems must dovetail with local data providers' processes and systems. Stand-alone systems and paper-based processes can no longer be considered adequate. An overarching consensus within this partnership is that business practices within state vital records offices and data providers must be documented and then updated to be more efficient and effective in light of today's technology and that these systems must be driven by national consensus-based standards and guidelines. The resulting reengineered state systems will use the 2003 version of the U.S. standard certificates of live birth, death, and fetal death. Reengineered systems will include efficient methods for capturing data, standard data-collection instruments, coding specifications, query guidelines, standardized definitions, and Health-Level-7–based standardized messaging. As the Public Health Information Network expands and is knitted together with a National Health Information Infrastructure, these reengineered vital statistics systems will need to be integrated with other health information systems, such as those for immunizations, newborn screening, and hearing screening, and with electronic systems used by data providers, including hospitals, physicians, and funeral homes.

The national partnership and its consensus process have already had some notable accomplishments, including the development of functional requirements for reengineered birth and death registration. The consensus national requirements will serve as the foundation for the design, development, and implementation of reengineered, Internet-based vital records and statistics systems for states. The most daunting challenge still to be overcome is the funding of the development and implementation of new systems, especially the automated reporting of deaths by the thousands of funeral directors and physicians who now manually provide mortality data.

Many questions are yet to be answered. What is the most effective way to retrieve quality medical information from the attending physician, coroner, or medical examiner? How can funeral directors and physicians be connected electronically and share with the state confidential information about the decedent in a secure environment? At what level of specificity do prompts and data edits for the medical information obtained from the physician become counterproductive? How can the state vital statistics systems take advantage of the data systems already in use by funeral directors and medical examiners? Efforts are currently underway to address these questions. Efforts to rejuvenate the nation's vital statistics system are encouraging, but they will need to expand dramatically to provide public health with a timely, high-quality, and flexible system to monitor vital health outcomes at the local, state, and national levels.

